# The Global Programme to Eliminate Lymphatic Filariasis: Health Impact after 8 Years

**DOI:** 10.1371/journal.pntd.0000317

**Published:** 2008-10-08

**Authors:** Eric A. Ottesen, Pamela J. Hooper, Mark Bradley, Gautam Biswas

**Affiliations:** 1 Lymphatic Filariasis Support Center, Task Force for Child Survival and Development, Decatur, Georgia, United States of America; 2 Global Community Partnerships, GlaxoSmithKline, Brentford, United Kingdom; 3 Preventive Chemotherapy and Transmission Control, World Health Organization, Geneva, Switzerland; University of Kelaniya, Sri Lanka

## Abstract

**Background:**

In its first 8 years, the Global Programme to Eliminate Lymphatic Filariasis (GPELF) achieved an unprecedentedly rapid scale-up: >1.9 billion treatments with anti-filarial drugs (albendazole, ivermectin, and diethylcarbamazine) were provided via yearly mass drug administration (MDA) to a minimum of 570 million individuals living in 48 of the 83 initially identified LF-endemic countries.

**Methodology:**

To assess the health impact that this massive global effort has had, we analyzed the benefits accrued first from preventing or stopping the progression of LF disease, and then from the broader anti-parasite effects (‘beyond-LF’ benefits) attributable to the use of albendazole and ivermectin. Projections were based on demographic and disease prevalence data from publications of the Population Reference Bureau, The World Bank, and the World Health Organization.

**Result:**

Between 2000 and 2007, the GPELF prevented LF disease in an estimated 6.6 million newborns who would otherwise have acquired LF, thus averting in their lifetimes nearly 1.4 million cases of hydrocele, 800,000 cases of lymphedema and 4.4 million cases of subclinical disease. Similarly, 9.5 million individuals—previously infected but without overt manifestations of disease—were protected from developing hydrocele (6.0 million) or lymphedema (3.5 million). These LF-related benefits, by themselves, translate into 32 million DALYs (Disability Adjusted Life Years) averted. Ancillary, ‘beyond-LF’ benefits from the >1.9 billion treatments delivered by the GPELF were also enormous, especially because of the >310 million treatments to the children and women of childbearing age who received albendazole with/without ivermectin (effectively treating intestinal helminths, onchocerciasis, lice, scabies, and other conditions). These benefits can be described but remain difficult to quantify, largely because of the poorly defined epidemiology of these latter infections.

**Conclusion:**

The GPELF has earlier been described as a ‘best buy’ in global health; this present tally of attributable health benefits from its first 8 years strengthens this notion considerably.

## Author Summary

Lymphatic filariasis (LF) is a vector-borne, chronically disabling parasitic infection causing elephantiasis, lymphedema, and hydrocele. The infection is endemic in 83 countries worldwide, with more than 1.2 billion people at risk and 120 million already infected. Since 1998, the Global Programme to Eliminate Lymphatic Filariasis (GPELF) has targeted elimination of LF by 2020. In its first 8 operational years, the program has scaled-up to provide more than 1.9 billion treatments through annual, single-dose mass drug administration (MDA) to ∼570 million individuals living in 48 LF-endemic countries. Not only do the GPELF drugs prevent the spread of LF, they also stop the progression of disease in those already infected. In addition, since two of the three drugs used for LF elimination have broad anti-parasite properties, treated populations are freed from both intestinal worms and from skin infections with onchocerca, lice, and scabies. To better understand the public health benefit of this ongoing global health initiative, we undertook an analysis of Programme data made available to WHO by participating countries. Our conservative estimates show that the GPELF has had an unprecedented public health impact on both LF and other neglected tropical diseases; it justly deserves the accolade of ‘a best buy’ in global health.

## Introduction

In 1997, the Global Programme to Eliminate Lymphatic Filariasis (GPELF) was created in response to a specific resolution by the World Health Assembly [1]. At that time the World health Organization (WHO), having recently devised a strategy aimed at achieving LF elimination through ‘mass drug administration’ (MDA) [2], received extraordinary pledges from two pharmaceutical companies (GlaxoSmithKline and Merck & Co., Inc.) for long-term drug donations of unprecedented size to jumpstart this nascent program.

The impressive *programmatic* progress made by the GPELF has been documented in a number of valuable reviews and updates [1], [3]–[7]; however, what is most needed now – for donors who are supporting this effort, for the Ministries of Health and health workers who are laboring on its behalf and for endemic communities who continue to invest their energies and resources towards its success – is to understand not just the technical achievements, but especially *what difference* it all has made to the health and welfare of the at-risk populations. What *impact* has 10 years of focus on LF – long recognized as one of the most debilitating and economically-draining of the neglected tropical diseases – really had?

To answer this question requires not just a tabulation of the GPELF's programmatic achievements in providing necessary drugs to the targeted at-risk populations, but also, importantly, a *projection* of the public health gain from this effort, using estimates based on the most accurate data and most reasonable assumptions available.

## Methods

### Data sources

Specific sources for the data are identified as they are presented; in general, however:

1. Numbers related to LF endemicity, populations at-risk (Table 1) and treatments delivered were derived from publications by WHO in the *Weekly Epidemiological Record (WER)* and WHO Annual Reports between 2000 and 2008 [4]–[10]; this information is also recorded at www.who.int/lymphatic_filariasis.
2. Information on the quantities of albendazole, ivermectin (Mectizan) and diethylcarbamazine (DEC) used in the GPELF came from these same *WER* reports [4]–[7], from WHO's Annual Reports (available at www.who.int/lymphatic_filariasis) and from records of GlaxoSmithKline and the Mectizan Donation Program.
3. Population demographic figures used to calculate age or gender subpopulations of the total at-risk populations were taken from the Population Reference Bureau [11] and the World Bank Health, Nutrition and Population Statistics [12].
4. Disability weights and formulas for calculating Disability Adjusted Life Years (DALYs) were derived from the *Global Burden of Disease* [13].
5. Information on the clinical profiles and the effectiveness of treatment for both LF and soil transmitted helminth (STH) infections has been taken from scientific publications [3], [14]–[16].
6. Estimates of the epidemiology of STH infections (number and distribution of affected individuals worldwide) came from published information [17].

**Table 1:** Population at Risk [5]

Region	# of Endemic Countries	At-Risk Population *(millions)*	Children at Risk *(millions)*
Africa (AFRO)	39	394	176
Americas (AMRO)	7	8.87	3.39
Eastern Mediterranean (EMRO)	3	14.9	6.50
South-east Asia (SEARO)	9	851	297
Western Pacific (WPRO)	25	31.6	11.1
**TOTAL:**	**83**	**1,300**	**494**

### Impact Projections

The assumptions made and the rationale behind the projections are outlined below and summarized in Tables 2 and 3.

**Table 2:** Projected Health Impact – LF Related.

Impact #1	Individuals Protected	Disease Prevented	DALYs Averted
	6.6 million newborns	1.4 million cases of hydrocele	3.2 million DALYs
		800,000 cases of lymphedema	2.8 million DALYs
		4.4 million cases of subclinical disease	?
	***Assumptions and Reasoning***		
	1) 66 million babies born into at-risk areas under MDA 2000–2007 (discounted for infant mortality) [11]		
	2) LF infections occur in 10% of at-risk population [3]		
	3) 12.5% of LF infections result in lymphedema, 20.8% in hydrocele, 66.7% in subclinical damage [3]		
	4) Disability weights (based on Global Burden of Disease methods): 0.105 for lymphedema, 0.073 for hydrocele; onset at age 20; life span is Region-specific		
	5) LF transmission (estimated by mosquito infection rates) falls progressively to 50%, 25%, 12%, 6%, and 0% pre-MDA levels after each of the first 5 MDAs, respectively		

Impact #2	Individuals Protected	Disease Prevented	DALYs Averted
	9.5 million people	6.0 million cases of hydrocele	14 million DALYs
		3.5 million cases of lymphedema	12 million DALYs
	***Assumptions and Reasoning***		
	1) 570 million individuals (at minimum) treated under MDAs 2000–2007. The maximal number of individuals treated in any single MDA was determined for each country. The sum of these numbers indicates the minimum total number of individuals treated.		
	2) LF infections occur in 10% of at-risk (i.e., treated) population [3] (here 57 million) with 1/3 having clinical manifestations and 2/3 having subclinical disease [3] (here 38 million)		
	3) To maintain this 1/3∶2/3 ratio 50% of those with subclinical disease must progress to overt disease (62.5% manifesting hydrocele [11.9 million] and 37.5%, lymphedema [7.1 million]) [3]		
	4) If treatment halts progression in only 50% of the subclinical cases (a conservative estimate [19]), 9.5 million people would have been protected from developing overt disease (6 million hydrocele; 3.5 million lymphedema)		
	5) Disability weights**: 0.105 for lymphedema, 0.073 for hydrocele; onset at age 20; life span, Region-specific		
	6) Treated individuals will not become re-infected in context of diminished LF transmission in MDA-covered areas		

**Table 3:** Projected Health Impact – Beyond LF.

Impact #3	Individuals Reached	Target	Health Benefits
	**56.6 million children** *-minimal estimate-*	Soil-transmitted helminthes (intestinal parasites: hookworm, roundworm, whipworm)	Weight/height gain, learning ability, cognitive testing, school attendance, fitness, activity [14], [26]–[28]
	***Assumptions and Reasoning***		
	1) 172 million treatments of albendazole given to children (age 2–15 in countries treated with DEC+albendazole; 5–15 in countries using ivermectin+albendazole) in 48 countries during MDAs 2000–2007 [4]–[7].		
	2) The maximal number of children treated *in any single MDA* was determined for each country. The sum of these numbers indicates the minimum total number of children treated (56.6 million) [4]–[7].		
	3) Uncertainty of STH prevalence estimates limits the specific quantification of health benefits despite their description in published studies [14], [26]–[28].		

Impact #4	Individuals Reached	Target	Health Benefits
	**44.5 million women of childbearing age** (not pregnant) *-minimal estimate-*	Soil-transmitted helminthes (intestinal parasites: hookworm, roundworm, whipworm)	Decreased anemia [16], maternal mortality, infant mortality; increased infant birth-weight [29]
	***Assumptions and Reasoning***		
	1) 140 million treatments of albendazole given to non-pregnant women-of-childbearing-age (15–49 years old) in 48 countries during MDAs 2000–2007 [4]–[7],[12].		
	2) The maximal number of such women treated in any single MDA was determined for each country [4]–[7]. The sum of these numbers indicates the minimum total number of women-of-childbearing-age treated (44.5 million).		
	3) Uncertainty of STH prevalence estimates limits the specific quantification of health benefits despite their description in published studies [16],[27],[30].		

Impact #5	Individuals Reached	Target	Health Benefits
	**45 million people in Africa** *-minimal estimate-*	Onchocerciasis, scabies, lice	Decreased physical, mental discomfort (severe itching) [32]; prevention of renal complications of streptococcal superinfections [35]
	***Assumptions and Reasoning***		
	1) 149 million treatments of ivermectin given to communities in 12 African countries during MDAs 2000–2007 [4]–[7].		
	2) The maximal number of individuals treated in any single MDA was determined for each country. The sum of these numbers indicates the minimum total number of individuals treated (45 million) [4]–[7].		
	3) Uncertainty of prevalence estimates for each of these conditions limits the specific calculation of health benefits despite the descriptions reported in published studies [32]–[34].		

#### Impact estimates: LF-related

*Babies protected from infection.* To estimate the number of babies born into LF treatment areas between 2000 and 2007, demographic data from each country (births per 1,000 population discounted by infant mortality rates [18] were applied to those populations living in areas targeted for LF treatments. Since LF transmission might not stop immediately after MDAs begin, changes observed in mosquito infection rates post MDA were used to estimate changes in LF transmission as progressively decreasing to 50%, 25%, 12%, 6%, and 0% of pre-MDA levels after each of the first 5 MDAs. These multipliers were used on a country-by-country and MDA-by-MDA basis to discount the number of surviving babies born into MDA areas, thereby allowing an estimate of the number of newborns protected from *potential* LF infection (66 million). Since LF infections are estimated to occur in approximately 10% of the at-risk population [3], 6.6 million newborn babies are therefore considered protected from contracting LF.

*Cases of morbidity prevented in newborns.* Globally, 12.5% of LF infections are estimated to result in lymphedema, 20.8% in hydrocele and the remainder, 66.7%, in subclinical disease [3]. Cases of disease averted (hydrocele, lymphedema and subclinical) were calculated by multiplying these proportions by the number of LF infections averted in babies.

*DALYs averted in newborns.* The number of DALYs averted in newborns was calculated using methods outlined in *Global Burden of Disease*, utilizing disability weights, the number of cases of clinical disease averted (hydrocele and lymphedema), an estimated onset of disease at age 20 and region-specific life spans [13]. Since disability weights are not available for subclinical LF disease, DALYs associated with this manifestation were not estimated.

For all of the calculations associated with the *prevention of LF disease*, it was assumed, based on available information, that treated individuals will not become re-infected in the context of diminished LF transmission in MDA-covered areas.

*Infected individuals protected from progression of subclinical disease to clinical disease.* For each country the number of individuals treated in each MDA is known, but since it is not known how many unique individuals have received treatment in a program with multiple MDAs, the conservative approach to identifying this number of unique individuals treated in any one country is to identify the maximal numbers of individuals treated in any single MDA for each country. These numbers were then summed for all countries and used as the *minimum* total number of individuals already treated (570 million). Since LF infections are estimated to occur in approximately 10% of the at-risk population [3], 57 million would be expected to be infected with LF. Approximately two-thirds of infected individuals have subclinical disease [3] (38 million), with 50% of *those* expected to progress to overt disease (19 million). Approximately 62.5% of individuals with overt disease manifest hydrocele (11.9 million) and 37.5% manifest lymphedema (7.1 million). If it is assumed that treatment halts disease progression in *only* 50% of subclinical cases (a conservative estimate [19]), 9.5 million people would have been protected from developing overt disease (*i.e.*, 6 million cases of hydrocele and 3.5 million cases of lymphedema averted).

*DALYs averted through halting progression of disease.* The number of DALYs averted through progression of disease was calculated using methods outlined in *Global Burden of Disease*, utilizing disability weights, the number of cases of clinical disease averted (hydrocele and lymphedema; calculated as described above), an estimated onset of disease at age 20 and region-specific life spans [13].

#### Impact estimates: ‘Beyond-LF’ benefits

Because individual country estimates of the prevalence and distribution of soil transmitted helminthiases are generally not available, it was not possible to estimate directly the number of STH infections, either in children or women of child bearing age, that have been treated as a consequence of LF MDA activities. However, since it is widely accepted that the common STH infections are distributed throughout the pan-tropical belt where lymphatic filariasis is endemic [17], we recognize that a proportion of the albendazole and ivermectin treatments delivered for LF will have had a beneficial impact for children and women of child bearing age who harbor intestinal helminth infections. The number of individual children less than 15 years of age treated with albendazole was estimated by multiplying demographic data (children under the age of 15 years, for each country [11] by that country's total treatment figures, then summing the maximal number of children treated in any single MDA for each country between 2000 and 2007 (the conservative estimate of the number of unique individuals treated; see above). Since age is an exclusion criterion for LF treatment, the annual estimates thus derived were discounted depending on the therapeutic regimen applied as follows: in ivermectin and albendazole areas of Africa and the Yemen, data for children 5 to 15 years of age only are included, whereas for the rest of the world where DEC and albendazole are utilized, data for children 3 to 15 years of age are included.

Women between 15 and 49 years were considered to be of childbearing age, and the number of individuals treated in this age class was calculated by multiplying demographic data [11] for each country by that country's total treatment figures, then summing the maximal number treated in any single MDA for each country between 2000 and 2007 (the conservative estimate of the number of unique individuals treated; see above). Since pregnancy is an exclusion criterion for LF treatment, the annual estimates thus derived were discounted by subtracting the estimated percent of the female population that is pregnant at any given time: the total fertility rate for each region was multiplied by a nine month gestational period and divided by 408 months (representing the estimated average number of reproductive months in a woman's lifetime).

Whilst the beneficial outcomes of treating STH infections in these population groups are listed, we do not attempt to *quantify* the accumulated health impact because of the uncertainty surrounding the prevalence estimates. The same rationale and argument adopted for soil transmitted helminth infections were applied when we considered the impact of ivermectin treatments on skin diseases of various etiology in Africa.

## Results

### Programmatic achievements of the GPELF 2000–2007

#### 1. The Global Programme

One hundred twenty million people are affected with LF – 40 million with limb or genital damage recognized as either lymphedema/elephantiasis (15 million) or hydrocele (25 million), and twice that number with subclinical disease principally of the lymphatics or kidneys [3]. These 120 million people live in 83 endemic countries of the tropics and subtropics where 1.3 billion people (1/5 of the world's population) comprise the total population considered ‘at risk’ for infection through their exposure to LF's mosquito-borne infective larvae (Table 1) [5]. More than a third of these are children [11].

Little more than a decade ago it was established that single doses of a 2-drug regimen (either albendazole+ivermectin or albendazole+DEC) can effectively eliminate microfilariae from the blood of infected individuals for periods often in excess of a year [20]. Once understood, this drug effectiveness permitted development of a strategy for LF elimination based on treating *entire* at-risk populations yearly with one of these two safe, effective 2-drug regimens in order to reduce microfilaremia (MF) below a ‘transmission threshold’ where future recrudescence would be unlikely even after population treatment was halted. From estimates of the life span of the adult parasites (*Wuchereria bancrofti* or *Brugia malayi*), from projections of the levels of ‘drug coverage’ that must be achieved in the targeted populations and from earlier experiences in countries targeting LF elimination, the average number of rounds of *effectively conducted*, yearly ‘mass drug administrations’ (MDAs) necessary to achieve success for national programs was estimated to be 4–6 [2]. Recent experience from both program observations and specific research studies is consistent with this notion that in most instances between 2 and 6 rounds of effective MDA are able to clear microfilaremia (see below for sentinel site data). There are, however, specific situations where more than 6 rounds might be required, since the number of MDAs necessary appears to depend principally on the pre-treatment microfilaremia levels, programmatic drug ‘coverage’ and local vector parasite complex [21].

#### 2. Treatments delivered

**Figure 1:**
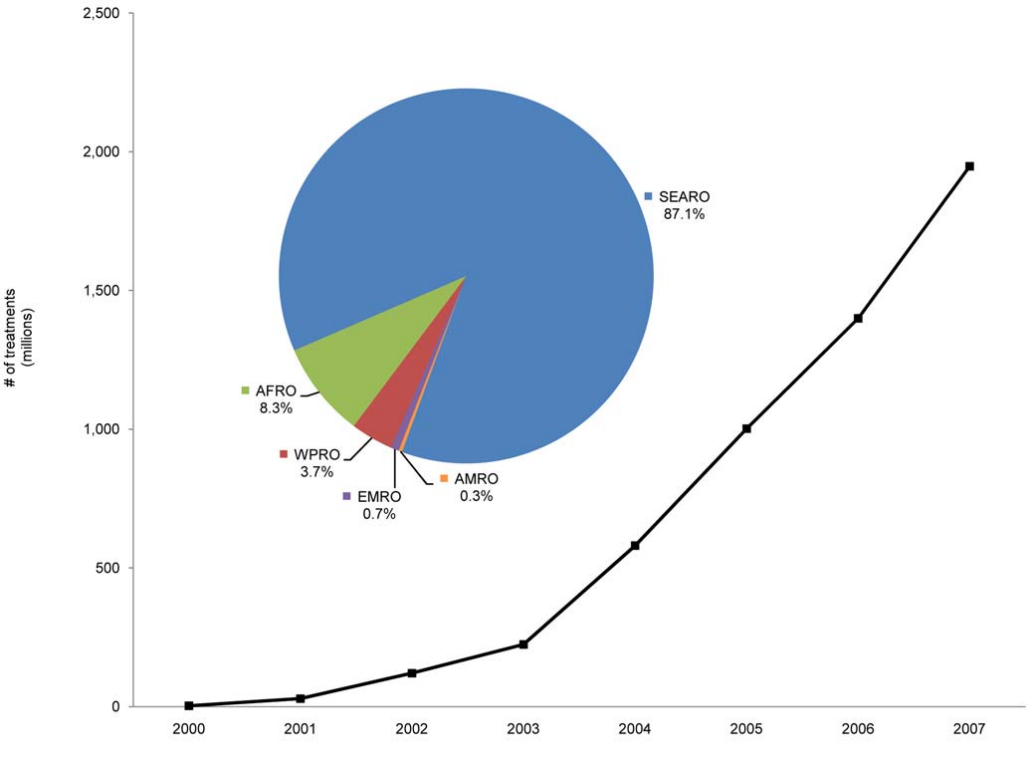
Cumulative treatments in GPELF. Progressive increase in number of treatments given through 2007; distribution by WHO region is depicted in pie-chart.

**Figure 2:**
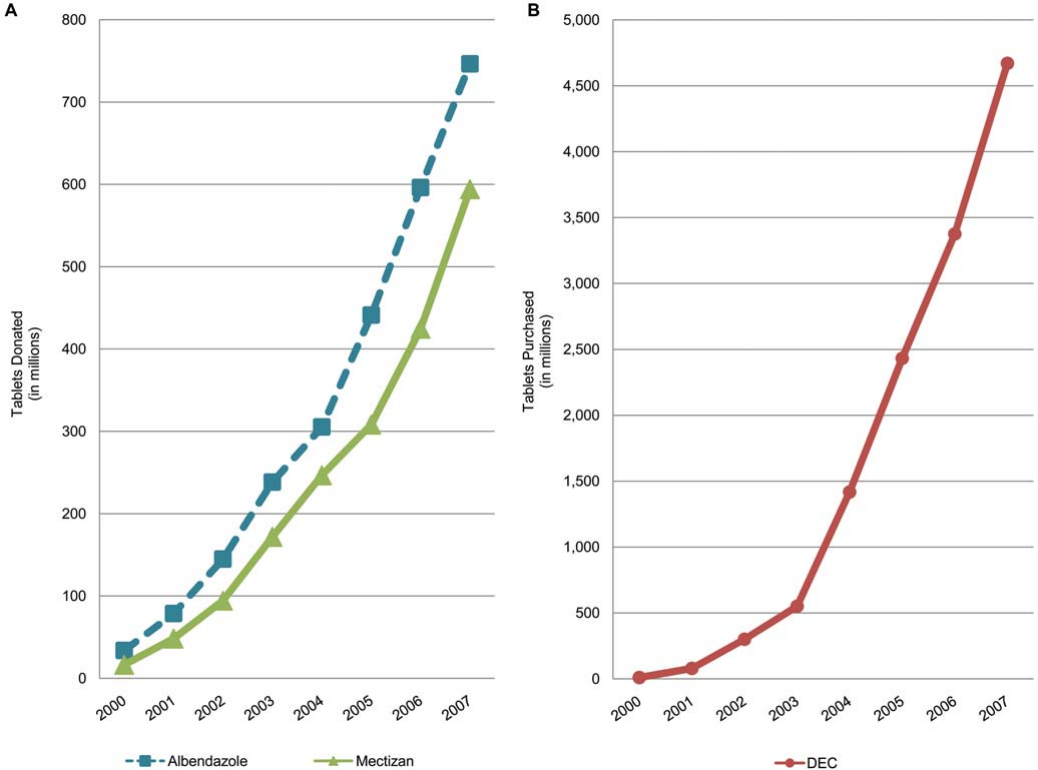
Cumulative totals of donated drugs (Panel A), albendazole and ivermectin (Mectizan), and purchased drug (Panel B) DEC, used in GPELF between 2000 and 2007.

Since its official inauguration in 2000 the GPELF has seen the most rapid expansion of any drug delivery program in public health history; by the end of 2007 more than *1.9 billion* treatments for LF had been delivered [7], almost ¾ by the program in India (initially a program based on DEC alone; more recently, on albendazole+DEC) with the remainder distributed in the 47 other countries with active MDA programs (Fig 1). The amount of drug *donated* to support this Programme has been extraordinary: more than 740 million tablets of albendazole and more than 590 million tablets of ivermectin were provided between 2000–2007 by the Global Programme's partners in the pharmaceutical industry. The amount of the non-donated drug (DEC) that had to be purchased during this same period by countries that utilize DEC instead of ivermectin (which is used for LF only in Africa [3]) was more than 4.7 *billion* tablets (Fig 2A & B).

#### 3. Programme effectiveness in decreasing LF prevalence

**Figure 3:**
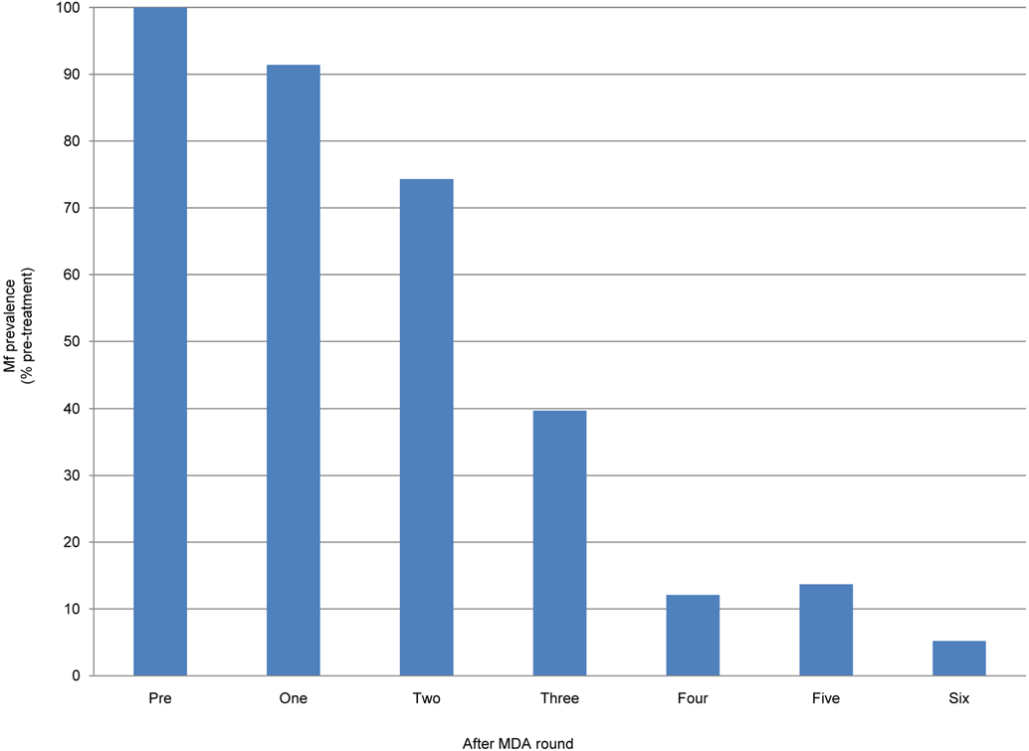
Effect of MDA on microfilaremia prevalence. Individuals in all of the sentinel sites (approximately 500 persons per site) reporting to the Global Programme were evaluated for microfilaremia. Progressive decline in prevalence among these individuals was recorded during yearly assessments (n = 131 sentinel sites for year 1; n = 124 for year 2; n = 139 for year 3; n = 148 for year 4; n = 68 for year 5; and n = 12 for year 6).

**Figure 4:**
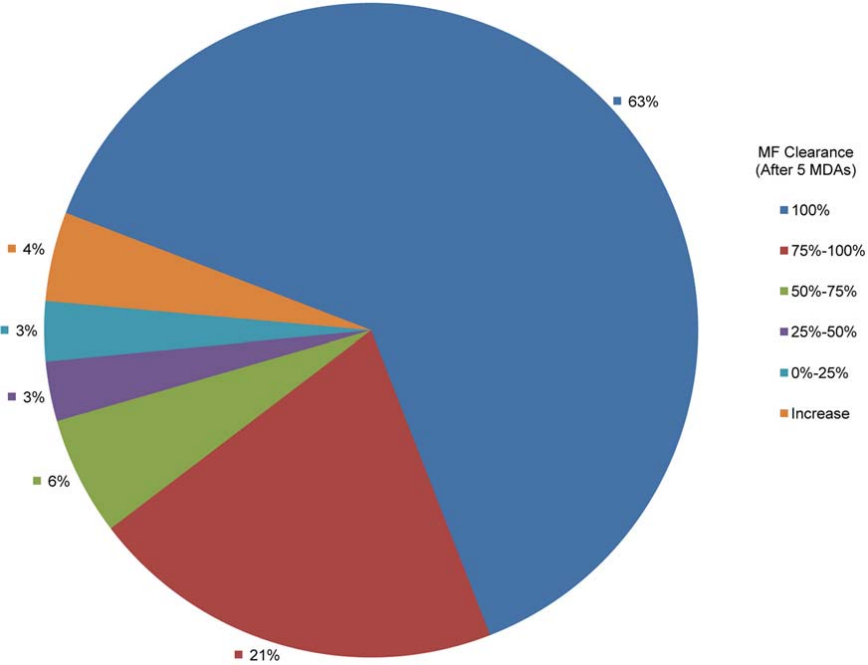
Clearance of microfilaremia from each sentinel site (approximately 500 persons per site) reporting to the Global Programme after 5 rounds of MDA treatment (n = 68).

The effectiveness of GPELF's strategy to reduce the prevalence of microfilaremia in an endemic population to levels below that believed necessary to sustain the parasite's life cycle has been substantiated by *research* teams in well-controlled, large-scale initiatives (e.g. in Egypt [22] and Papua New Guinea [23]). In addition, assessment of *programmatically* collected data available to WHO from another 20 countries shows similar progressive declines in mf prevalence in treated communities (Fig. 3), with greater than 10-fold reduction in mf-prevalence levels seen in sentinel-site communities that have received 6 rounds of MDA and *total* clearance of mf (by inference, interruption of LF transmission) recorded in almost 2/3 of the communities after 5 MDA rounds (Fig. 4).

### Health impact of the GPELF 2000–2007

As impressive as the record is for the number of treatments given, the number of albendazole and ivermectin tablets donated, the amount of DEC purchased, and the number of communities cleared of microfilaremia during the first 8 years of this Global Programme, still *the* most important Programme outcome is the overall *health* benefit that the GPELF has brought to populations at-risk for LF. This benefit must derive from *projections* based on the best data and most reasonable assumptions available (see below and Tables 2 & 3 for the assumptions and implications).

There are two principal sources of this health benefit:

1. LF-related benefits – *i.e.*, those coming directly from the effects of the MDAs in preventing the acquisition of lymphatic filarial disease or in arresting its progression
2. ‘Beyond-LF’ benefits – *i.e.*, those coming from ancillary benefits of the highly effective, broad-spectrum anti-parasitic drugs, albendazole and ivermectin, used in the Programme.

#### 1. Projected health impact that is LF-related

*Protecting newborns from LF infection and disease.* Since MDAs, by decreasing and then stopping LF transmission, will prevent uninfected individuals from becoming infected, the clearest measure of the Programme's long-term health impact is the amount of disease prevented over the lifetime of babies born into areas where their likelihood of acquiring infection has become much diminished or nil. To determine this impact requires an understanding of the number of babies born (and surviving) in areas covered by LF MDAs, the number who would have acquired infection (and disease) in the absence of GPELF, the ‘disability weights’ for different manifestations of LF disease and the rate at which exposure to LF infection declines in treated populations. When these variables were assessed [see Discussion and Table 2 for fuller description], the following conclusions could be made:

- ***Impact #1 - Prevention of LF infection (and disease):*** *Between 2000–2007, 6.6 million newborns (the fraction of all newborns who would have been expected to acquire LF) were protected by GPELF – thereby averting in their lifetimes nearly 1.4 million cases of hydrocele, more than 800,000 cases of lymphedema and 4.4 million cases of subclinical disease (* *Table 2* *).*
-
  - *Because of this disease prevention, 6.0 million Disability Adjusted Life Years (DALYs) have been averted (3.2 million from prevention of hydrocele and 2.8 million from prevention of lymphedema* [Table 2]).

*Preventing the progression to overt disease in LF-endemic populations.* With evidence now available that the MDA treatment regimens for LF can halt, or even reverse, the progression of subclinical to overt disease [19],[24],[25], it is clear that those already infected but having no overt disease also benefit directly from the yearly MDAs. To quantify this benefit requires understanding the number of *individuals* treated during the MDAs, the proportion of these individuals with subclinical LF disease, the number who would have progressed to each of the manifestations of LF disease and the ‘disability weights’ for each of these manifestations. When all of these were considered (see Discussion and Table 2), the following could be recognized:

- ***Impact #2 - Prevention of LF disease:*** *Between 2000–2007, 9.5 million individuals – previously infected but without overt manifestations of disease – were protected by GPELF from* developing *hydrocele (6.0 million) or lymphedema 3.5 million)*.
-
  - *This disease prevention translates into 26 million DALYs averted (14 million from hydrocele prevention and 12 million from lymphedema prevention).*

#### 2. Projected health impact from ‘Beyond-LF’ benefits

*Preventing the consequences of intestinal parasite infections*. The best drugs to control intestinal parasites (*i.e.*, ‘soil-transmitted helminths’ [STH]: hookworm, roundworm and whipworm) are the same drugs (albendazole and ivermectin) used to eliminate LF [3],[20]. Though Mectizan (ivermectin) has formal regulatory approval only for lymphatic filariasis and onchocerciasis and is donated by Merck & Co., Inc. only for those indications, each year millions of children and women-of-childbearing-age are concomitantly treated for debilitating *intestinal parasite* infections (without additional cost or effort) while participating in their national programs to eliminate *LF*. To identify the impact of such treatment requires estimation of the number of children and the number of women-of-childbearing-age who received albendazole (with or without ivermectin) in all GPELF countries. Thus,

- ***Impact #3 - ‘Beyond-LF’ benefit for children with intestinal parasites:*** *Between 2000–2007, more than 172 million treatments for intestinal parasite infections were given to 56.6 million children by GPELF (* *Table 3* *)*
-
  - *Based on earlier research studies, each infected child receiving treatment would be expected to develop increased appetite* [*26*] *(leading, in some settings, to 1 kg of extra weight gain and 0.6 cm extra growth in the first 5 months)* [*27*] *; greater eye-hand coordination, learning ability and concentration* [*14*] *; better school attendance, cognitive testing (20% improvement)* [*28*], *fitness scores and spontaneous play activity (43% increase)* [*26*],[*27*].
- ***Impact #4 - ‘Beyond-LF’ benefit for women-of-childbearing-age with intestinal parasites:*** *Between 2000–2007 more than 140 million treatments for STH were given to 44.5 million women-of-childbearing age by GPELF (* *Table 3* *).*
-
  - *Repeated treatment of hookworm and other intestinal parasites improves both nutritional status and, most importantly, iron stores in women during their reproductive years* [*16*],[*29*] *. Prior studies predict that such treatment can lead to an increase in infant birth-weights by more than 50 grams and a drop in infant mortality by as much as 40%* [*29*] *.* Maternal *mortality should also decrease significantly in women receiving GPELF treatments, since iron deficiency anemia is a prominent cause of maternal mortality* [*30*] *.*

*Prevention of debilitating skin diseases.* Onchocerciasis, scabies, and pediculosis (lice) are all diseases of the skin caused by parasites common in resource poor communities and associated with appreciable mental and physical disability in affected populations. Ivermectin, one of the two drugs co-administered by the GPELF in Africa, is the best oral treatment for all of these debilitating skin diseases [31]–[33]; it is also the mainstay drug for onchocerciasis control programs in Africa [34]. To gauge the GPELF impact on skin diseases it is necessary first to understand the number of individuals receiving ivermectin through GPELF activities in Africa. Thus,

- ***Impact #5 - ‘Beyond-LF’ benefit for people with skin diseases in Africa:*** *Between 2000–2007, over 149 million treatments with ivermectin were administered by GPELF or APOC (African Programme for Onchocerciasis Control) to more than 45 million people in African communities (* *Table 3* *) where the prevalence of scabies skin infection may exceed 30% and the prevalence of onchocerciasis even more.*
-
  - *Ivermectin's long lasting impact on scabies can cause community prevalence to fall dramatically after 1 cycle of treatment and to disappear almost completely after 2 or more treatments* [*31*] *. Cured individuals show improvements in sleep patterns and overall wellbeing, but also importantly, treatment of scabies in childhood can prevent the post-streptococcal renal disease induced by group B streptococcus skin infections that often complicate chronic scabies infection* [*35*] *.*
  - *Because of its broad geographic range, the GPELF has brought ivermectin treatment to* additional millions of people *living in onchocerciasis-endemic areas not previously targeted by onchocerciasis control programs (as these programs focus only on communities where the prevalence of onchocerciasis exceeds 40%)* [*34*] *.*

## Discussion

Since WHO's Global Programme to Eliminate Lymphatic Filariasis was officially launched in 2000, its *programmatic* achievements [recorded here through 2007] are unparalleled (Box 1): 1.9 billion treatments delivered through yearly MDAs to over 570 million people in 48 endemic countries. These accomplishments were made possible by the enormous drug donations of albendazole (over 740 million tablets from GlaxoSmithKline through 2007) and ivermectin (over 590 million tablets of Mectizan from Merck & Co., Inc.), by the willingness of National Programs to procure 4.7 billion tablets of DEC, and by the early support from numerous other organizations – most significantly the Bill and Melinda Gates Foundation, the Arab Fund for Economic and Social Development, the international development agencies of Japan and the United Kingdom and the Ministries of Health of endemic countries.

**Box 1. The Global Programme to Eliminate LF – Its First 8 Years.**

**Reach** Nearly 2 billion treatments delivered to more than 560 million people in 48 countries.

**Dissemination** More than 50% of endemic countries actively involved in annual MDA programmes.

**Child Protection** Nearly 176 million children already treated for LF, and over 66 million babies born into areas now protected by MDA.

**Public Health Impact on LF** More than 6 million cases of hydrocele and 4 million cases of lymphoedema prevented, translating into more than 32 million DALYs averted.

**Additional Health Benefits** More than 310 million treatments of albendazole delivered to women of child-bearing age and school-age children, providing sustained relief from the negative consequences of soil-transmitted helminth (STH) infections that include maternal anemia, low birth weight newborns, excess infant mortality, inhibited growth and development, diminished intellectual performance.

Almost 150 million treatments of ivermectin delivered to African communities, providing sustained relief from onchocercal skin disease, scabies, lice and important STH infections.

Though it is without question that this Programme has had a very great impact on global health, quantifying this impact still poses difficult challenges. Principally this is because all projections must be made not just from the *numbers* of people treated but also from the more-difficult-to-quantify *effects* of such treatment. Assumptions derived from current best understanding must be linked with the available data to formulate the health impact projections, and while making such assumptions is never entirely satisfactory, the present analysis does endeavor to identify clearly both the assumptions themselves and the sources of the data used to generate the projections; it also has chosen to err on the conservative side in most estimations.

For the GPELF, health benefits lie in two domains: one related to the Programme's effects on lymphatic filarial disease and its consequences, and the other related to the outcome of treating LF-endemic populations with one or both of the very safe, broad-spectrum anti-parasitic drugs used by the Programme, albendazole and ivermectin.

### LF-related impact

To gauge the LF-related impact, this analysis has considered quantitatively only what has been accomplished by: 1) preventing infection in those born into areas where GPELF is active and 2) stopping the progression to clinical disease in previously infected individuals whose disease has not yet expressed itself overtly.

1) To identify the amount of infection prevented, the number of babies born in areas under LF MDAs between 2000–2007 who survived infancy was first determined, by country [11],[12]. Estimation of how many of these newborns would have acquired LF during their lives and what manifestations they would have developed was based on the global prevalence figures available for LF and its clinical manifestations (Table 2) [3]. Calculation of the DALYs attributable to that amount of disease during the lifetimes of those newborns assumed that clinical expression of disease (hydrocele and lymphedema) had its onset at an average age of 20 years and persisted throughout the life of the individual.

Since the risk of exposure of these infants to LF depends on the level of local transmission, it is necessary to estimate the rate of decline of transmission (here using vector infection in mosquitoes as a surrogate for transmission) as MDA programs progress. While programmatic evidence exists that effective *transmission* of LF might cease very soon after the initiation of MDA activities [22],[23],[36], entomologic studies linked with anti-filarial single-dose treatment regimens indicate that the decline in *vector infection* may be more gradual [22], [23], [37]–[41]. Since the availability of such data is too limited (with respect to vector species, collection techniques, parasite assessments, LF prevalence, treatment regimens, and other variables) to give *precise* estimates of post-MDA changes in vector infection, data from available studies [22], [23], [37]–[41] were pooled, yielding a relationship that describes an ‘average’ rate-of-decline of vector infection; namely, declines to 50%, 25%, 12%, 6% and 0% of pre-treatment levels following each of the first 5 MDAs, respectively. (As these numbers were empirically defined, they already incorporate the influence of population ‘coverage’ on MDA effectiveness.) This information was then used to estimate the effect that each MDA had for each treated population in each country in order to approximate the exposure to LF in infants born after initiation of GPELF activities.

2) Stopping the progression of subclinical to clinical disease in those already infected contributes appreciably to the calculations of LF-related health benefits from GPELF (Table 2). Evidence for such effectiveness of MDA regimens in halting disease progression is relatively recent and has focused particularly on children with subclinical or early-stage lymphatic disease [19],[25]. Because these effects are just now being studied comprehensively, and in order to be conservative in estimating GPELF's health impact, the present calculations are based on the conservative assumption [19] that the MDA programs would arrest subclinical disease progression in *only 50%* of the affected individuals (Table 2).

Though one cannot be completely certain of all of the calculations in Table 2, it is still hard to escape the conclusion that these values for GPELF's *LF-related* health impact are almost certainly gross underestimates – for at least 2 reasons. First, not considered at all in the assessments of GPELF's *LF-prevention benefits* are those related to any of the manifestations of LF disease other than hydrocele and lymphedema. Among those omitted, quantitatively most important would be the Programme's impact on subclinical LF disease [24],[25],[42] – especially microfilaremia, hematuria, lymphatic dilatation and lymphatic dysfunction – which affect a very large percentage of those with LF infection [3] but for which there are no ‘disability weights’ available for calculating DALYs or DALYs averted. Also overlooked are other extremely important, often debilitating *overt* clinical manifestations of infection – especially, the very common, recurrent acute adenolymphangitis episodes (ADL) and the progressive, crippling pulmonary disease, tropical pulmonary eosinophilia (TPE) [3]. Excluding all of these important consequences of LF infection from the calculations of GPELF's health impact from preventing LF ensures that these calculations will significantly underestimate the Programme's impact.

Second, none of these quantitative calculations of GPELF's LF-related health impact has taken into consideration the *direct* effect that this Programme has had on arresting progression or ameliorating clinical disease of affected individuals. In addition to its delivery of essential anti-filarial drugs, the GPELF is also a program that advocates and initiates ‘morbidity management’ activities based on vigorous personal hygiene management of lymphedema or elephantiasis [43]. Dramatic improvement in both physical state and mental attitude occurs in patients following the hygiene guidelines [43],[44], but none of the health impact of this component of the GPELF has been quantified or captured in the calculations of Table 2. Similarly uncaptured is the potential direct improvement in both lymphedema and hydrocele now being reported by patients following MDA treatment alone (*i.e.*, even in the absence of hygiene management) [23].

### ‘Beyond-LF’ Health Impact

If the *LF-related* health impact of GPELF seems difficult to quantify, the ‘beyond-LF’ impact presents an even greater challenge. A major reason is that many of the ‘beyond LF’ benefits come from the impact that the GPELF drugs have on soil transmitted helminth (STH) infections in the treated populations. The quantitative epidemiology of these infections remains poorly characterized, albeit for good reasons: not only are STH infections caused by *three* distinct parasites (hookworm, roundworm and whipworm), but these three infections also occur in unequal proportions in different endemic regions and cause different diseases with varying severity and health consequences. Further, while the geographic overlap of STH infections with the LF at-risk areas is felt to be almost universal [45], it is rarely known which STH infections occur or with what abundance in which areas. Thus, while general estimates of overall STH prevalence can be approximated for areas where GPELF is active, the data itself is not certain enough to be used quantitatively to project GPELF's health impact from treating STH infections.

Despite such limitations, a number of very important studies *have* been carried out to document and measure the health consequences of STH infections – usually by monitoring changes in outcome indicators following treatment with albendazole or other drugs. These have shown, for example, that

1. Soil transmitted helminth infections exact a severe toll on the nutritional status and growth of infected children, but intervention with albendazole and ivermectin can make an extraordinary difference in their physical development, with spectacular gains in growth parameters quantified in a number of important studies [14]–[16],[46],[47].
2. Lethargy and lack of physical stamina often characterize children infected with intestinal worms, but within weeks of treatment significant increases can be found in physical activity and spontaneous play. Resting heart rates, physical fitness on the Harvard step test, and measurements of spontaneous play behavior all improved in children from Kenya and Indonesia after being treated for intestinal worms [14],[26],[27],[47].
3. Children infected with intestinal worms are frequently seen to miss many more school days than their uninfected peers, as documented in Jamaica where children with intense Trichuris infections missed twice as many school days as their infection-free peers [48]. Treatment leads to significant reduction in school absenteeism; a 25% reduction was recorded in Kenya following school-based treatment for STH [49].
4. Children infected with intestinal worms perform poorly in learning ability tests, cognitive function and educational achievement, but treating school age children increases their ability to learn, as documented by improvement in children's short and long term memory, executive function language, problem solving and attention [50],[51].

These STH infections that are treated by the GPELF MDAs are not just important for children. While their effect on the health and productivity of men remains poorly defined, in women-of-childbearing-age hookworm infection is recognized as a major cause of anemia, and this anemia significantly affects both maternal and newborn morbidity and mortality. Indeed,

1. WHO estimates that women in developing countries may be pregnant for half their reproductive lives and are at an increased risk of anemia during this time [30].
2. Anemia in pregnancy has been clearly associated with poor birth outcome, including low birth-weight [52]–[55] and increased maternal morbidity and mortality [30],[56],[57].
3. Hookworm-attributable anemia, induced by deficiencies in iron, protein and total energy, is a significant cause of intrauterine growth retardation and low birth weight [58]. It might even exacerbate the sometimes fatal effects of malaria infection in infants and young children.
4. Treating STH infections in women-of-child-bearing-age improves both maternal health status and the status of infants born to infection-free mothers; therefore, WHO recommends that anthelminthic treatment be included in strategies to improve maternal nutrition wherever hookworm infection and anemia are prevalent [30]. (GPELF, however, currently restricts its treatment to women who are not pregnant.)

In addition to its effect on certain of the STH infections, ivermectin – as GPELF's second drug with broad-spectrum anti-parasite activity – is unsurpassed for the oral treatment of both onchocerciasis [34] and ectoparasites (scabies and lice) [31]. While ivermectin has been the mainstay of onchocerciasis control programs for the past 2 decades, the control programs in Africa (where 99% of the onchocerciasis is found) have as their principal target only communities designated hyper- or meso-endemic (*i.e.*, prevalence ≥40%), so that many communities endemic for onchocerciasis were left untreated until GPELF was initiated [34]. Since LF is distributed very much more widely than onchocerciasis, and since almost all regions of Africa where onchocerciasis is endemic are also ‘at risk’ for LF, GPELF activity in those areas has resulted in the treatment of millions of additional individuals in these onchocerciasis-endemic areas who were not covered under the older control programs. These individuals are generally not those with blinding onchocerciasis but with severe onchocercal skin disease (OSD) and “troublesome itching”; the burden of illness from this OSD, quantified in DALYS lost, is recognized as essentially equivalent to that estimated for onchocercal ocular disease and blindness [33]. GPELF's impact on improving OSD is not yet quantified, but it *can* be defined once the number of individuals with onchocerciasis who live in the expanded treatment areas is more well understood [34]. On the other hand, for the very important skin diseases caused by scabies and lice, the significant health benefits that GPELF brings through its use of ivermectin in affected populations will be much more difficult to quantify, since so much less is known about the epidemiology of these widespread ectoparasite diseases, and no burden-of-illness estimates have yet been established [32].

The Global Programme to Eliminate LF is not a static program; indeed, its reach continues to expand each year. In 2008 it is projected that >500 million people will be treated in that year alone. The effect on the *calculated* health benefits of the Programme that these progressively increasing numbers will have each year is enormous, since the number of protected children and cases of disease prevented will increase rapidly as new cohorts of treated individuals are added each year; in addition, of course, all of those benefits not currently quantified (both LF-related and beyond-LF effects) will continue to multiply as well.

Already the GPELF has been described as a ‘best buy’ in global health, and the present tally of health benefits only strengthens this contention. Even during its first 8 years, almost 2 billion MDA treatments have been given and 32 million DALYs-averted have been identified by considering (conservatively) just 2 of the 5 specific impacts attributable to the Programme (Tables 2 & 3). Considering only these DALYs and estimating treatment costs at $0.10/person (a ‘high’ estimate given the fact that the preponderance of treatments were in countries where costs have been identified as being much lower [59]) suggests that, excluding the donated drug costs, $190 million will have been spent to effect the 1.9 billion treatments. If the 32 million averted DALYs were the *only* benefits achieved, each DALY averted by the Programme would have cost $5.90. This cost is extremely low compared to DALY averted costs of other programs [60], but even *it* is a gross overestimate of the true cost of DALYs-averted by GPELF activities, since so much of the Programme's benefit (Tables 2 & 3) remain unquantified and not included in this calculation. As this LF Elimination Programme continues to expand, its benefits will continue to accrue; as our ability to quantify these benefits improves, the Programme's true value will become progressively still more impressive.

## Supporting Information

Alternative Language Abstract S1 Translation of the Abstract into French by P. J. Hooper
(0.06 MB PDF)
