# Supplementary material for: The Global Programme to Eliminate Lymphatic Filariasis: Health Impact after 8 Years
Source: PLoS Negl Trop Dis. 2008 Oct 8;2(10):e317. doi: 10.1371/journal.pntd.0000317 (PMC2556399; doi:10.1371/journal.pntd.0000317)
Supplement: Alternative Language Abstract S1 — Translation of the Abstract into French by P. J. Hooper (0.06 MB PDF) [file pntd.0000317.s001.pdf]

The Global Programme to Eliminate Lymphatic Filariasis: Health Impact after 8 Years

Eric A. Ottesen[1], Pamela J. Hooper[1], Mark Bradley[2], Gautam Biswas[3]

[1] Lymphatic Filariasis Support Center, Task Force for Child Survival and Development, Decatur, GA, USA, [2] Global Community Partnerships, GlaxoSmithKline, Brentford, UK, [3] Preventive Chemotherapy and Transmission Control, World Health Organization, Geneva, Switzerland

Contexte : Pendant ses premières 8 années, le Programme Mondial pour l'Élimination de la Filariose Lymphatique a connu un accroissement rapide sans précédent : plus de 1,9 milliards de traitements contre la filariose lymphatique avec l'albendazole, l'ivermectine et la diethylcarbamazine ont été fournis à travers la distribution massive de médicaments annuelle à un minimum de 570 millions d'individus vivant dans 48 des 83 pays initialement identifiés comme étant endémiques pour la FL.

Méthodologie : Pour évaluer l'impact sanitaire de cet important effort mondial, nous avons analysé les bénéfices cumulés d'abord de la prévention ou l'arrêt de la progression de la filariose lymphatique, puis des effets antiparasitaires plus larges (les bénéfices 'au-delà de la FL') attribuables à l'utilisation de l'albendazole et de l'ivermectine. Les projections ont été faites sur la base des données démographiques et de prévalence de la maladie dans les publications du Population Reference Bureau, la Banque Mondiale et l'Organisation Mondiale de la Santé.

Résultats : Entre 2000 et 2007 le Programme Mondial a permis d'éviter la filariose chez environ 6,6 millions de nouveau-nés qui auraient en principe contracté la FL – leur évitant ainsi presque 1,4 millions de cas d'hydrocèle, 800.000 cas de lymphoedème et 4,4 millions de cas d'atteintes infra-cliniques. De même, 9,5 millions d'individus – précédemment infectés mais sans manifestations visibles de la maladie – ont été protégés contre le développement d'une hydrocèle (6,0 millions) ou d'un lymphoedème (3,5 millions). Ces bénéfices liés au traitement de la FL équivalent à 32 millions années de vie corrigées du facteur d'invalidité (DALY) évitées.

De même, les bénéfices auxiliaires 'au-delà de la FL' résultant de plus de 1,9 milliards de traitements délivrés par le Programme Mondial ont été énormes, particulièrement grâce à plus de 310 millions de traitements administrés aux enfants et femmes en âge de procréer qui ont reçus l'albendazole avec ou sans l'ivermectine (traitant efficacement les helminthiases d'origine tellurique, l'onchocercose, les poux, la gale et autres maladies). Ces bénéfices peuvent être décrits mais restent difficiles à quantifier, en grande partie à cause de l'insuffisance dans l'épidémiologie des dernières infections ci-dessus citées.

Conclusion : Le Programme Mondial a antérieurement été décrit comme le ' meilleur achat' en matière de santé ; ce calcul actuel des bénéfices sanitaires attribuables à ses premières 8 années renforce considérablement cette notion.
